# Dipolar cations confer defect tolerance in wide-bandgap metal halide perovskites

**DOI:** 10.1038/s41467-018-05531-8

**Published:** 2018-08-06

**Authors:** Hairen Tan, Fanglin Che, Mingyang Wei, Yicheng Zhao, Makhsud I. Saidaminov, Petar Todorović, Danny Broberg, Grant Walters, Furui Tan, Taotao Zhuang, Bin Sun, Zhiqin Liang, Haifeng Yuan, Eduard Fron, Junghwan Kim, Zhenyu Yang, Oleksandr Voznyy, Mark Asta, Edward H. Sargent

**Affiliations:** 1 Department of Electrical and Computer Engineering, University of Toronto, 35 St. George Street, Toronto, ON M5S 1A4 Canada; 2 National Laboratory of Solid State Microstructures, Collaborative Innovation Centre of Advanced Microstructures, Jiangsu Key Laboratory of Artificial Functional Materials, College of Engineering and Applied Sciences, Nanjing University, 210093 Nanjing, Jiangsu China; 3 Department of Materials Science and Engineering, University of California, Berkeley, CA 94720 USA; 4 Materials Sciences Division, Lawrence Berkeley National Laboratory, Berkeley, CA 94720 USA; 5 Key Lab of Photovoltaic Materials, Department of Physics and Electronics, Henan University, 475004 Kaifeng, China; 6 Department of Chemistry, KU Leuven, Celestijnenlaan 200 F, B-3001 Leuven, Belgium

## Abstract

Efficient wide-bandgap perovskite solar cells (PSCs) enable high-efficiency tandem photovoltaics when combined with crystalline silicon and other low-bandgap absorbers. However, wide-bandgap PSCs today exhibit performance far inferior to that of sub-1.6-eV bandgap PSCs due to their tendency to form a high density of deep traps. Here, we show that healing the deep traps in wide-bandgap perovskites—in effect, increasing the defect tolerance via cation engineering—enables further performance improvements in PSCs. We achieve a stabilized power conversion efficiency of 20.7% for 1.65-eV bandgap PSCs by incorporating dipolar cations, with a high open-circuit voltage of 1.22 V and a fill factor exceeding 80%. We also obtain a stabilized efficiency of 19.1% for 1.74-eV bandgap PSCs with a high open-circuit voltage of 1.25 V. From density functional theory calculations, we find that the presence and reorientation of the dipolar cation in mixed cation–halide perovskites heals the defects that introduce deep trap states.

## Abstract (web summary)

The performance of wide-bandgap perovskite photovoltaics is limited by the undesired phase transition and high density of deep level traps. Here, Tan et al. incorporate dipolar methylammonium cation to make the material defect-tolerant and achieve a high power conversion efficiency of 20.7%.

## Introduction

Wide-bandgap perovskite solar cells (PSCs) enable efficient monolithic tandem devices with crystalline silicon (c-Si) and other leading low-bandgap materials because of their high open-circuit voltage (*V*_oc_) and tunable bandgaps (*E*_g_)^1–7^. As the front subcell, the wide-bandgap PSCs should simultaneously exhibit high *V*_oc_ (low *V*_oc_ deficit), high fill factor (FF), and sufficient photocurrent density to match the bottom junction.

Varying halide composition enables perovskites with wide bandgap of around 1.7 eV^8^, optimal for tandem cells with c-Si and other absorbers having about 1.1 eV bandgap^9–11^. Stable wide-bandgap perovskites have been achieved by using mixed cations of formamidinium (FA) and cesium (Cs) on the A-site of the perovskite structure^12,13^. Using a composition of Cs_0.17_FA_0.83_Pb(I_0.6_Br_0.4_)_3_, Snaith et al. obtained a stabilized power conversion efficiency (PCE) of around 16% and a maximum *V*_oc_ of 1.2 V in 1.74-eV perovskite solar cells^12^. Performance improvements in wide-bandgap PSCs were made by enlarging the grain size^14^, reducing interfacial traps^15^, adding rubidium^16^, and improving surface treatments^17^. Despite these advances, wide-bandgap PSCs still exhibit modest PCEs, large *V*_oc_ deficits (*E*_g_–*V*_oc_), and low FF, leading to performance well inferior to that of sub-1.6 eV bandgap PSCs^18–21^.

The poor performance of wide-bandgap PSCs is limited by a high trap density in the polycrystalline perovskite absorber. We reasoned that healing the deep traps in perovskite absorber—in effect, increasing the defect tolerance^22,23^—could enable further performance improvements in wide-bandgap PSCs.

Sub-1.6 eV bandgap PSC strategies indicate that A-site cation compositional engineering is crucial to achieve the best-performing devices^18,20,24,25^. Specifically, the most efficient PSCs contain a small amount of methylammonium (MA) cation. The mixing of MA and FA has primarily been utilized to achieve enhanced structural stability in FAPbI_3_-based perovskites^26^.

The electronic role—if one exists—played by the MA cation in the mixed cation-lead halide perovskites that led to the best-performing PSCs has seen initial studies of considerable interest in MAPbI_3_ perovskites; this important topic is much less studied in mixed cation perovskites.

In single cation MAPbX_3_ (X = Cl, Br, or I) perovskites, the collective rotation of dipolar MA cations has been proposed to enhance charge transport due to the formation of large polarons or the spatial localization of carriers^27–29^. The liquid-like reorientation of MA cations has been found to protect hot carriers in MAPbX_3_ perovskites^30,31^. The dipolar disorder of MA cations has been found to contribute to high dielectric constants in MAPbX_3_ perovskites, which should assist in screening of charged states or carriers^32^. Recent computational work has also revealed that deep trap states in MAPbI_3_ perovskite can be healed by dynamic rotation of MA cations in response to point charges^33^.

Inspired by these studies, we examine the impact of the MA cation on the electronic properties of FA-Cs-based mixed cation–halide wide-bandgap perovskites. We fabricate wide-bandgap PSCs and significantly boost the photovoltaic performance by incorporating a small amount of MA additive. We achieve a stabilized PCE of 20.7% in 1.65-eV bandgap PSCs, with a high *V*_oc_ of 1.22 V and a FF exceeding 80%. The performance improvement is also validated for 1.74-eV bandgap PSCs, giving a stabilized PCE of 19.1% together with a high *V*_oc_ of 1.25 V. Using computational studies, we see a role of the MA cation in mixed perovskites, wherein the presence and the reorientation of dipolar MA cation eliminates—or renders innocuously shallow—electronic states associated with key defects. These states, in the absence of MA, would lead to deep traps.

## Results

### Characterization of wide-bandgap perovskite films

**Fig. 1:**
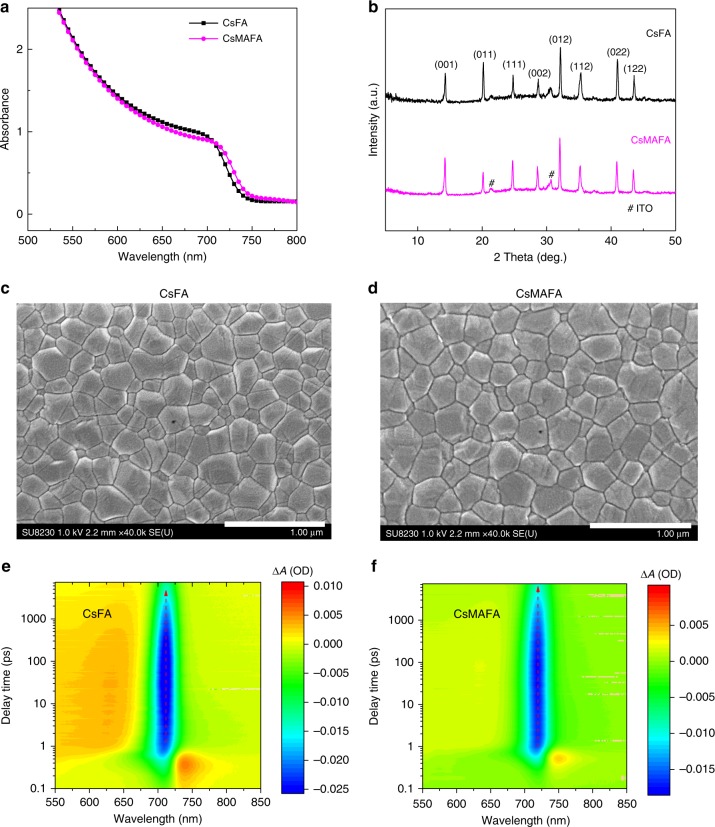
Optical and structural characterization of wide-bandgap perovskite films. **a** Absorbance of perovskite films, corresponding to absorption onset of 1.67 eV for CsFA and 1.65 eV for CsMAFA. **b** XRD patterns of CsFA and CsMAFA perovskite films on ITO/TiO_2_–Cl substrates. **c**–**d** SEM images of CsFA and CsMAFA perovskite films on ITO/TiO_2_–Cl substrates. The scale bars are 1 μm. **e**–**f** Transient absorption studies of CsFA and CsMAFA films on glass substrates. The vertical red dashed lines indicate the peak position of photobleaching band over a probe delay time of 1–7000 ps. OD optical density

We fabricated wide-bandgap perovskite thin films with and without (controls) the dipolar MA cation (see Methods). Both compositions—Cs_0.2_FA_0.8_Pb(I_0.75_Br_0.25_)_3_ (denoted CsFA) and Cs_0.05_MA_0.15_FA_0.8_Pb(I_0.75_Br_0.25_)_3_ (denoted CsMAFA)—exhibit a sharp absorption edge (*E*_g,abs_) at 1.67 and 1.65 eV, respectively (Fig. 1a). The absorption onset, rather than Tauc plot bandgap, is used here due to excitonic contributions to the spectrum close to the bandgap^8,15^ (Supplementary Fig. 1 and Supplementary Table 1). The partial replacement of Cs with MA shifts the absorption onset to slightly longer wavelength. Nuclear magnetic resonance (NMR) spectroscopy results further confirm that the ratios of MA and FA organic cations in the precursor solutions and the CsMAFA perovskite films are consistent (Supplementary Figs. 2 and 3 and Supplementary Table 2). We chose to experiment with perovskites having bandgaps of 1.65–1.70 eV, since previous modeling indicates that such bandgaps are ideal for top cell when monolithically combined with a c-Si real cell under practical conditions^9,34^.

X-ray diffraction (XRD) studies of films reveal a single cubic phase without PbI_2_ and non-perovskite yellow phases in each case (Fig. 1b). The XRD peak positions of CsMAFA film shift to slightly lower diffraction angles because of the larger ionic size in the case of MA, compared to Cs. Adding MA cations does not measurably affect the surface morphology. Both CsFA and CsMAFA films, each having a thickness around 600 nm, are smooth and dense without noticeable pinholes. The two sets of films exhibit comparable grain size and similar grain growth (Fig. 1c, d and Supplementary Fig. 4).

Photoinduced phase segregation is a critical concern for wide-bandgap perovskites, given its detrimental effect on the stability of device performance under operation^12,35,36^. We carried out femtosecond transient absorption (TA) spectroscopy to further check the single phase as indicated by XRD and to investigate the photostability of CsMAFA perovskite films. Each sample was subjected to illumination using a 400-nm pump at a fluence of 4 µJ cm^−2^ (average pump power density of 20 mW cm^−2^) for at least 30 min in ambient air during TA measurements. Both CsFA and CsMAFA perovskite films showed photobleaching peaks that remained consistent at 711 and 719 nm, respectively (Fig. 1e, f). We also checked the TA spectra of CsMAFA perovskite film at several different excitation wavelengths (Supplementary Fig. 5) and found no shift in the bleaching peak. These results indicate that wide-bandgap perovskite with CsMAFA triple cations is single phase in composition and phase stable under light illumination.

### Improved photovoltaic performance with dipolar cation

**Fig. 2:**
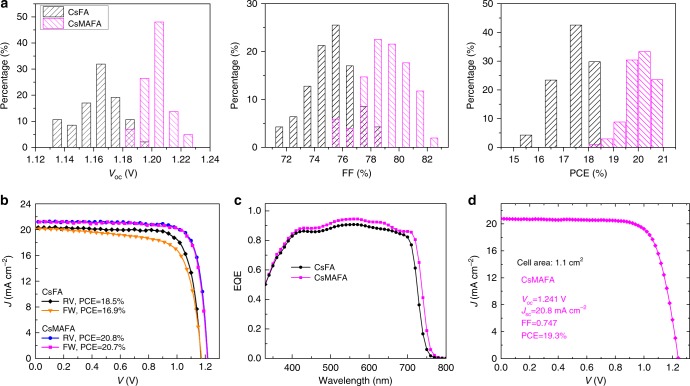
Photovoltaic performance of wide-bandgap perovskite solar cells. **a** Histograms of *V*_oc_, FF, and PCE of 47 CsFA solar cells and 102 CsMAFA devices. **b** Reverse (RV) and forward (FW) *J*–*V* curves of best-performing CsFA and CsMAFA perovskite solar cells. **c** EQE curves of CsFA and CsMAFA perovskite solar cells, corresponding to integrated *J*_sc_ values of 19.4 and 20.7 mA cm^−2^, respectively. **d** *J*–*V* curve of best-performing CsMAFA solar cell with an active area of 1.1 cm^2^. CsFA and CsMAFA stand for the compositions of Cs_0.2_FA_0.8_Pb(I_0.75_Br_0.25_)_3_ and Cs_0.05_MA_0.15_FA_0.8_Pb(I_0.75_Br_0.25_)_3_, respectively

We fabricated planar PSCs using the device architecture of ITO/TiO_2_–Cl/perovskite/Spiro-OMeTAD/Au (Supplementary Fig. 4). We deposited a thick perovskite layer (about 600 nm) in order to ensure substantially complete light harvesting above the bandgap. Chlorine-capped TiO_2_ (TiO_2_–Cl) nanocrystal films were used to form the electron selective layer for contact passivation^18^. Figure 2a presents the statistical photovoltaic performance of PSCs using CsFA and CsMAFA perovskites measured under reverse scans. The CsMAFA PSCs exhibit considerably better performance than CsFA devices for all metrics. The average *V*_oc_ increases by 50 mV from 1.16 ± 0.02 to 1.21 ± 0.01 V. The average FF increases from 76 ± 2 to 79 ± 2%. The average *J*_sc_ increases from 19.9 ± 0.5 to 21.0 ± 0.4 mA cm^−2^, mainly due to the slightly lower bandgap of CsMAFA. Correspondingly, the average PCE increases from 17.5 ± 0.8 to 20.0 ± 0.5%.

The photovoltaic performances of the best-performing CsFA and CsMAFA perovskite solar cells are shown in Fig. 2b and Table 1. The CsMAFA devices exhibit a best stabilized PCE of 20.7% (Supplementary Fig. 6) and negligible hysteresis, whereas the CsFA devices have considerable hysteresis, in agreement with previous studies^12,14,17^. The integrated *J*_sc_ values from EQE spectra (Fig. 2c) are consistent with the *J*−*V* measurements (within 5% deviations). The high FF values that exceed 80% and low *V*_oc_ deficit in CsMAFA devices are comparable to those of the best-reported lower-bandgap (below 1.6 eV) PSCs^18–20^. The CsMAFA wide-bandgap PSCs achieved here exhibit a low *V*_oc_ deficit, sufficiently large *J*_sc_, and high FF and thus match well with high-efficiency c-Si solar cells^37^ needed in the future realization of monolithic tandem devices. To explore film uniformity, we fabricated large-area CsMAFA PSCs with an active area over 1 cm^2^ (Fig. 2d). The devices exhibit a maximum PCE of 19.3% with a low *V*_oc_ deficit (*E*_g_−*V*_oc_) of 410 mV. We tracked the operating stability of CsFA and CsMAFA perovskite solar cells at their maximum power point (MPP) conditions under one sun illumination (Supplementary Fig. 7). The CsMAFA solar cells have stable *V*_oc_ under illumination, and they also exhibit comparable operating stability to the case of CsFA and maintain more than 97% of their initial efficiencies following continuous MPP operation for 11 h.

**Table 1:** Photovoltaic performance of best-performing wide-bandgap perovskite solar cells with and without MA cation

Composition	*E*_g,abs_ (eV)	Scan direction	*V*_oc_ (V)	*J*_sc_ (mA cm^−2^)	FF (%)	PCE (%)	Stabilized PCE (%)
Cs_0.2_FA_0.8_Pb(I_0.75_Br_0.25_)_3_	1.67	RV	1.17	20.4	77.3	18.5	17.6
		FW	1.17	20.2	71.3	16.9	
Cs_0.05_MA_0.15_FA_0.8_Pb(I_0.75_Br_0.25_)_3_	1.65	RV	1.22	21.2	80.5	20.8	20.7
		FW	1.22	21.3	79.9	20.7	
Cs_0.17_FA_0.83_Pb(I_0.6_Br_0.4_)_3_	1.74	RV	1.22	18.7	75.6	17.2	16.7
		FW	1.21	18.4	70.5	15.7	
Cs_0.12_MA_0.05_FA_0.83_Pb(I_0.6_Br_0.4_)_3_	1.74	RV	1.25	19.0	81.5	19.3	19.1
		FW	1.25	19.0	80.0	19.0	

*E*_g,abs_: absorption onset, RV: reverse scan, FW: forward scan

### Reduced recombination loss in MA-containing perovskites

**Fig. 3:**
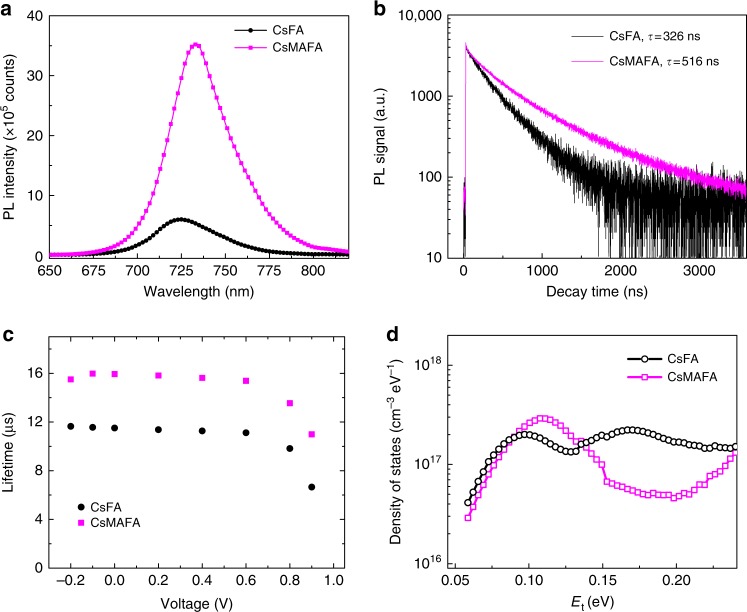
Reduced trap density in MA-containing wide-bandgap perovskites. **a** Steady-state photoluminescence (PL) spectra and **b** time-resolved PL decay curves of CsFA and CsMAFA perovskite films deposited on glass substrates. **c** Recombination lifetimes of CsFA and CsMAFA solar cells extracted from impedance spectroscopy. **d** Trap density of states obtained from thermal admittance spectroscopy for CsFA and CsMAFA perovskite solar cells

We then turned to the study of the mechanistic origins of the performance improvement that arose upon the incorporation of MA into wide-bandgap PSCs. We used steady-state photoluminescence (PL), time-resolved PL (TR-PL), and impedance spectroscopy to study the charge recombination kinetics of perovskite films. The PL emission intensity of CsMAFA perovskite films is five times higher than that of CsFA samples (Fig. 3a). This indicates that adding MA cation reduces the non-radiative recombination sites in the perovskite film. To test the possibility that enhanced PL emission achieved by adding MA cations is mainly due to the change in film surface or grain boundaries, we spin-cast a thin layer of tri-n-octylphosphine oxide (TOPO) ligands on the as-obtained perovskite films with the goal of achieving surface/grain passivation, as demonstrated in previous reports^38,39^. Both CsFA and CsMAFA perovskite films exhibited similar PL enhancements (Supplementary Fig. 8), indicating that the reduced non-radiative recombination in the CsMAFA perovskite films can be attributed principally to the improved bulk quality achieved by adding MA cations. We further carried out the TR-PL decays of perovskite films (Fig. 3b). The PL decay lifetime of CsMAFA film (516 ns) is longer than that of CsFA (326 ns), consistent with a reduced trap density in CsMAFA and suppressed non-radiative recombination channels.

We carried out thermal admittance spectroscopy (TAS) measurements to examine the trap density of states (*t*_DOS_) in CsFA and CsMAFA perovskite solar cells. TAS probes shallow and deep trap states in solar cells^40–42^. The CsMAFA solar cells exhibited longer recombination lifetime than CsFA devices (Fig. 3c), implying slower charge recombination rate in CsMAFA. Figure 3d presents the *t*_DOS_ of CsFA and CsMAFA perovskite solar cells measured under illumination at short-circuit condition. For CsFA devices, there are two obvious peaks: one, trap-state distribution with the peak position at 0.095 eV and another, deeper trap-state distribution with the peak position at 0.18 eV. The integrated trap densities of the shallow and deep trap levels are 0.6 × 10^16^ cm^−3^ and 2.1 × 10^16^ cm^−3^, respectively. For CsMAFA devices, however, there is only one shallow trap-state distribution with the peak position at 0.11 eV, corresponding to an integrated trap density of 1.1 × 10^16^ cm^−3^.

### Performance improvement in 1.74-eV bandgap solar cells

We further explored the impact of MA incorporation on the photovoltaic performance of other wide-bandgap PSCs. In ideal case (Shockley–Queisser limit for each junction), an optical bandgap of 1.75 eV is desired for the top cell, in a tandem with c-Si. In this context, perovskite composition of Cs_0.17_FA_0.83_Pb(I_0.6_Br_0.4_)_3_ has a near-ideal bandgap of 1.74 eV^12^. By incorporating 5% MA (a composition of Cs_0.12_MA_0.05_FA_0.83_Pb(I_0.6_Br_0.4_)_3_), we obtained perovskites having a substantially unaltered bandgap compared with Cs_0.17_FA_0.83_Pb(I_0.6_Br_0.4_)_3_ (Supplementary Fig. 9). Stronger PL emission and longer PL decay lifetime were observed in the MA-containing perovskite films. Both *V*_oc_ and FF of solar cells increased significantly after incorporation of MA (Table 1 and Supplementary Fig. 10a). *J*_sc_ increases slightly as well, mainly due to enhanced charge collection in the long wavelength spectral region (Supplementary Fig. 10b). Consequently, stabilized PCE increases considerably from 16.7 to 19.1%. The hysteretic behaviors in *J*–*V* measurements are substantially eliminated upon MA incorporation. FF exceeds 80% and *V*_oc_ increases to 1.25 V after the incorporation of a small amount of MA. The *V*_oc_ deficit (490 mV) is higher than that of 1.65-eV bandgap devices (410 mV). Increasing the grain size and optimizing the interfaces may further reduce the *V*_oc_ deficit^14,15^.

### Defect healing by dipolar cations

We now explore how the incorporation of MA cation could reduce the trap-mediated recombination in mixed cation–halide perovskites. In MAPbX_3_ perovskites, the dynamic motion of MA cation was proposed to facilitate the formation of polarons^28^. Since the large effective mass of the polaron can shield the carriers from impurity scattering, polaron formation may reduce carrier scattering by defects or phonons and protect the hot carriers as well as bandedge carriers^28–30^. This has been offered as one important reason for the high-defect tolerance in perovskites. However, the formation of large polarons is predominantly associated with the deformation of the PbX_3_^−^ framework (irrespective of the A-site cation type)^43,44^. From transient absorption spectra, we found that, in the CsFA and CsMAFA systems, the films exhibit a comparable polaron formation time of about 0.4 ps, consistent with previously reported value (Supplementary Fig. 11a)^43,44^. We propose that the small MA additive concentration (5–15%) used herein does not quantitatively impact the polaronic effect. We also carried out quasi-elastic neutron scattering (QENS) experiments to query the motion of cations in CsFA and CsMAFA perovskites (Supplementary Fig. 11b). The results indicate that CsFA and CsMAFA perovskites exhibit cation dynamic motion^45–47^.

One crucial difference between MA and FA/Cs cations is the much higher dipole moment for MA (about 2.3 *D*) compared with FA (about 0.2 *D*) and Cs (non-polar) due to their different molecular configuration (Supplementary Fig. 12)^48^. The liquid-like reorientation of dipolar MA cations has been found to protect hot carriers in MAPbX_3_ perovskites^30,31^. The dipolar disorder of MA cations has been found to contribute to high dielectric constants in MAPbX_3_ perovskites, which could assist in the screening of charged states or carriers (reducing the defect trapping cross-section)^32^. Recent computational work has also revealed that deep trap states in MAPbI_3_ perovskite can be healed by dynamic rotation of MA cations in response to point charges^33^.

While it is challenging to experimentally observe the reorientation of MA in response to defect sites and correlate the motion of MA cation with trap levels, we carried out density functional theory (DFT) calculations to explore how the MA cation influences the electronic properties of FA-Cs-based wide-bandgap perovskites (see Methods). We compared the materials (with composition close to our experimental materials) having vs. lacking the MA cation within these two formulae: Cs_0.2_FA_0.8_ PbI_2_Br (referred to as CsFA) and Cs_0.05_MA_0.15_FA_0.8_PbI_2_Br (referred to as CsMAFA) (see Supplementary Fig. 13).

We sought first to explore the impact of MA incorporation on the defect formation energies of the Schottky-type vacancy defects and charge-balanced antisite defects (Supplementary Fig. 14 and Supplementary Note 1). The partial incorporation of MA (15% MA) leads to slightly lower defect formation energies, except in the case of the Pb–I antisite defect. We concluded that MA incorporation does not reduce the formation of defects.

We then proceeded to investigate how the presence and dynamic motion of dipolar MA cations close to the defect site affects the electronic properties of the defects that give rise to deep trap states in CsFA perovskite, antisite defects Pb_I_, Br_Pb_, and I_Pb_, and interstitial defect Pb_i_^49–51^. Here, we investigated reorientation of the dipolar MA cation. We investigated the effects that arise due to the fact that the dipole moment of MA is much stronger than that of FA, and that the kinetic barrier to the reorientation of FA is ten times higher than that of MA^48^. Compared to the MA cation, the FA cation has a negligible electrostatic effect in the presence of charges (free carriers or charged defects).

**Fig. 4:**
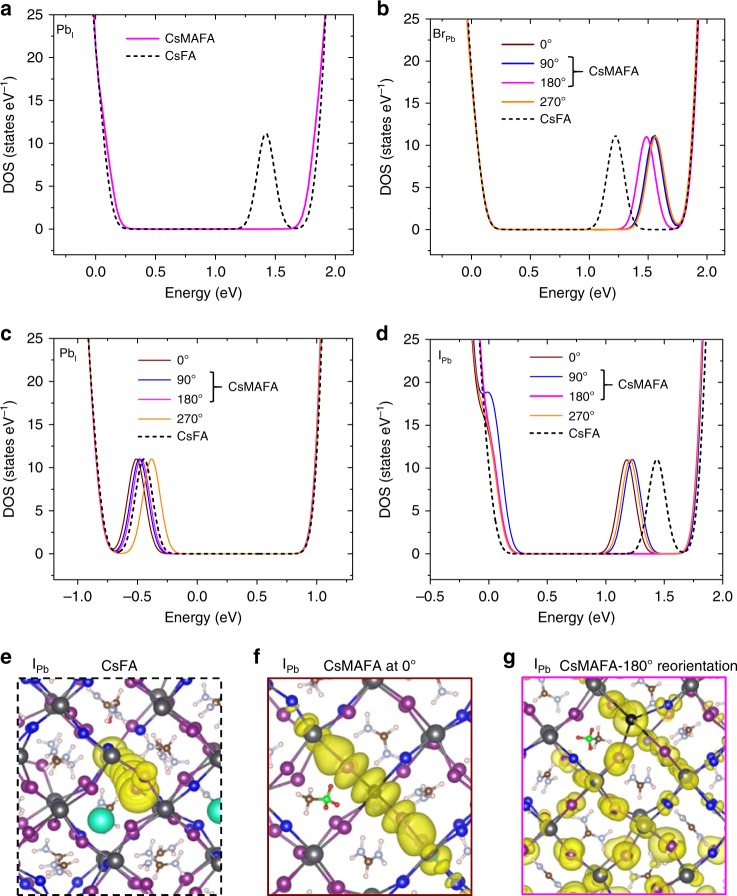
Effects of MA reorientation on the electronic properties of charged defects. **a** Density of states (DOS) of CsFA and CsMAFA perovskites in the case of defect (Pb_I_) that in-gaps states are present in CsFA perovskite, but not in CsMAFA perovskite, regardless of the reorientation direction. **b**–**d** Density of states (DOS) of CsFA and CsMAFA perovskites in the cases of defects (Br_Pb_, Pb_i_, and I_Pb_) that introduce in-gap states in both perovskites, but the reorientation of MA cation renders shallower trap levels. **e**–**g** Wave functions of perovskites with the *I*_Pb_ defect: CsFA perovskite (**e**) and CsMAFA perovskite when the MA cation is randomized at 0^o^ (**f**) or is reoriented at the lowest energy direction 180^o^ (**g**)

We investigated the first scenario, wherein the defect (Pb_I_) introduces in-gap trap states only in the CsFA perovskite (Fig. 4a). We found that, following MA incorporation, such in-gap states disappear in the CsMAFA perovskite, irrespective of the orientation direction of the MA cation.

In the second scenario, the defect (Pb_i_, Br_Pb_, and I_Pb_) introduces in-gap states in both CsFA and CsMAFA perovskites, but the reorientation of MA cation near the defect site renders shallower trap states (Fig. 4b–d). For the Br_Pb_ defect, MA incorporation with various MA reorientations consistently shifts the deep trap states to much shallower levels near the CBM (Fig. 4b). For the Pb_i_ defect, with a certain MA rotation scenario (orange line in Fig. 4c), CsMAFA may have deeper traps than CsFA. As the MA cation rotates into more energetically favored orientations (Supplementary Table 3), the trap states shift to shallower levels than those in CsFA. In case of I_Pb_ defect, it introduces deeper trap states in CsMAFA than CsFA when MA orientation is randomized (Fig. 4d). As the MA cation reorients to its lowest energy (most energetically favored) direction (180^o^, pink line in Fig. 4d, NH_3_^+^ facing opposite to the defect), the deep trap states shift to very shallow levels near the VBM. The charge densities derived from the wave functions associated with the I_Pb_ defect states in CsFA and CsMAFA perovskites are visualized in Fig. 4e–g. When the defect introduces deep trap states (Figs. 4e, f), the wave functions are localized near the charged defect site. After the MA reorients to its lowest energy direction, the trap states become very shallow near the VBM (Fig. 4d) and the wave function becomes delocalized (Fig. 4g), suggesting a reduced impact on carrier trapping.

## Discussion

Our DFT studies reveal that the incorporation and reorientation of the dipolar MA cation in mixed perovskites offer the potential to heal the defects that introduce deep trap states in CsFA perovskite. The computational findings agree well with our experimental results, which show much reduced non-radiative recombination with MA incorporation. The trap-assisted Shockley–Read–Hall (SRH) recombination rate *R*_SRH_ can be expressed as Eq. (1) by assuming same capture cross-sections for electrons and holes^52^:

1 $$R_{{\mathrm{SRH}}} = V_{{\mathrm{th}}}\sigma N_{\mathrm{t}}\frac{{np - n_{\mathrm{i}}^2}}{{n + p + 2n_{\mathrm{i}}{\mathrm{cosh}}\left( {\frac{{E_{\mathrm{t}} - E_{\mathrm{i}}}}{{k_{\mathrm{B}}T}}} \right)}}$$

where *σ* is capture cross-section of the traps, *n*_i_ is the intrinsic carrier concentration, *N*_t_ is trap density, *E*_t_ is trap energy level, *n* is electron concentration, *p* is hole concentration, *k*_B_ is Boltzmann constant, and *T* is absolute temperature. The recombination rate *R*_SRH_ can be reduced in CsMAFA perovskite by decreasing the capture cross-section of traps due to the screening of defects by MA dipoles, or by reducing in-gap trap density or rendering shallower trap levels (schematically shown in Supplementary Fig. 15).

Charge carrier screening from (charged) defects enabled by MA is an overall consequence of the interactions between free carriers, charged defects, and dipoles. Charged defects generate a local electric field that attracts free carriers with opposite polarity and thus promotes trap-assisted SRH recombination. When a dipolar cation is in the proximity of a charged defect site, the dipolar cation reorients in response to the local electrical field due to the electrostatic interaction. This reduces the charge seen by free carriers and thus reduces the capture cross-section for non-radiative recombination. This screening effect depends on the direction of the dipole near the charged defect. As seen in our DFT studies, the in-gap trap states are closely related to the reorientation direction. When the local electric field $\left( {{\vec{\mathbf F}}} \right)$ induced by charged defect is aligned with the dipole moment $\left( {{\vec{\mathbf d}}} \right)$, the change in energy ${{U}}$ can be expressed as $\left( { - {\vec{\mathbf d}} \cdot {\vec{\mathbf F}}} \right)$. We show that when the energy of hybrid halide perovskites is lowered by MA reorientation, the trap level becomes shallower (and the wave function becomes delocalized).

We investigated various defect types, including antisite, interstitial, and vacancy defects. We find that, for antisite and interstitial defects, the reorientation of the dipolar methylammonium (MA) cation in mixed perovskites heals the defects that introduce deep trap states (Fig. 4). We also investigated the vacancy defects that do not introduce in-gap trap states in CsFA and CsMAFA perovskites (Supplementary Fig. 16). Taking the I vacancy (*V*_I_) as an example, we report that the reorientation of MA does not change the defect behavior in this case (Supplementary Fig. 17).

We note that our ground-state simulations are not without their limitations. As shown by Nan et al., trap-inefficient defects in the ground state (e.g., Pb and halide vacancies) may become trap-efficient defects in the excited state^33^. This means that those defects for which the orientation of MA is irrelevant in the ground state (e.g., Pb and I vacancies) may become deep traps following excitation. We expect that the dynamic motion of dipolar cation may also contribute to healing defects that only act as traps in the excited state. Mixed perovskites require a large number of atoms in DFT simulations, and our currently available computational capacity only allows us to carry out ground-state simulations. Further DFT studies (when larger computational capacity will be accessible) that take account of the excited-state conditions will enable further insights into dipolar cations and their role in defect healing in mixed cation–halide perovskites.

In summary, the incorporation of dipolar MA cation in photostable CsFA-based wide-bandgap perovskites significantly boosts solar cell efficiency by reducing the trap-assisted non-radiative recombination. The incorporation of MA does not necessarily reduce the formation of defects, but the incorporation of the dipolar MA cation in mixed cation–halide wide-bandgap perovskites heals deep trap defects, resulting in a more defect-tolerant material. These findings shed light on defect healing in perovskite materials and pave the way to further increasing the efficiency of perovskite-enabled tandem photovoltaic devices.

## Methods

### Materials

Unless stated otherwise, all materials were purchased from Sigma-Aldrich or Alfa-Aesar and used as received without further purification. The organic halide salts (MABr, FAI, and FABr) were purchased from Dyesol Inc., Australia.

### Solar cell fabrication

The pre-patterned indium tin oxide (ITO, TFD Devices)-coated glass was sequentially cleaned using acetone and isopropanol. A chlorine-capped TiO_2_ (TiO_2_–Cl) nanocrystal electron transport layer (around 60 nm in thickness) was spin-coated twice on ITO substrate at 3000 rpm for 30 s from the colloidal TiO_2_–Cl nanocrystal solution (5 mg mL^−1^). The TiO_2_–Cl solution was prepared according to previous work^18^. The TiO_2_–Cl film was then annealed on a hot plate at the displayed temperature of 150 °C for 30 min in ambient air. After the substrates had cooled, we immediately transferred the substrates to a nitrogen-filled glovebox for the deposition of perovskite films. The perovskite precursor solutions (1.4 M) were prepared in a mixed solvent of DMF and DMSO (volume ratio 4:1). The molar ratios of 1  mL solution are listed as follows. Cs_0.2_FA_0.8_Pb(I_0.75_Br_0.25_)_3_: 0.07 mmol CsI, 0.21 mmol CsBr, 1.12 mmol FAI, 0.98 mmol PbI_2_, and 0.42 mmol PbBr_2_; Cs_0.05_MA_0.15_FA_0.8_Pb(I_0.75_Br_0.25_)_3_: 0.07 mmol CsI, 0.21 mmol MABr, 1.12 mmol FAI, 0.98 mmol PbI_2_, and 0.42 mmol PbBr_2_; Cs_0.17_FA_0.83_Pb(I_0.6_Br_0.4_)_3_: 0.07 mmol CsI, 0.168 mmol CsBr, 0.77 mmol FAI, 0.392 mmol FABr, 0.84 mmol PbI_2_, and 0.56 mmol PbBr_2_; Cs_0.12_MA_0.05_FA_0.83_Pb(I_0.6_Br_0.4_)_3_: 0.07 mmol CsI, 0.098 mmol CsBr, 0.07 mmol MABr, 0.77 mmol FAI, 0.392 mmol FABr, 0.84 mmol PbI_2_, and 0.56 mmol PbBr_2_. The perovskite films were deposited onto the TiO_2_–Cl substrates with a two-step spin-coating procedure. The first step was 2000 rpm for 10 s with an acceleration of 200 rpm s^−1^. The second step was 6000 rpm for 40 s with a ramp-up of 2000 rpm s^−1^. Chlorobenzene (100 µL) was dropped on the spinning substrate during the second spin-coating step at 20 s before the end of the procedure. The substrate was then immediately transferred to a hot plate and heated at 100 °C for 30 min. After cooling down to room temperature, the hole-transport layer was subsequently deposited on top of the perovskite film by spin coating at 3000 rpm for 30 s using a chlorobenzene solution, which contained 72.3 mg mL^−1^ of Spiro-OMeTAD and 28.8 µL mL^−1^ of tert-butylpyridine, as well as 17.0 µL mL^−1^ of bis(trifluoromethane)sulfonimide lithium salt (520 mg mL^−1^ stock solution in acetonitrile). Finally, 100-nm Au contact was deposited on top of Spiro-OMeTAD by electron beam evaporation in an Angstrom Engineering deposition system.

### Solar cell characterization

The current density–voltage (*J*–*V*) characteristics were measured using a Keithley 2400 sourcemeter under the illumination of a solar simulator (Newport, Class A) at the light intensity of 100 mW cm^−2^, as checked with a calibrated reference solar cell (Newport). Unless otherwise stated, the *J*–*V* curves were all measured in nitrogen atmosphere with a scanning rate of 50 mV s^−1^ (voltage step of 10 mV and delay time of 200 ms). Spectral mismatch factor of 1 was used. The steady-state PCE was measured by setting a bias voltage to *V*_MPP_ and then tracing the current density. *V*_MPP_ at maximum power point was determined from the reverse *J*–*V* curve. The active area was determined by the aperture shade mask (0.049 cm^2^ for small-area devices and 1.1 cm^2^ for large-area devices) placed in front of the solar cell to avoid overestimation of the photocurrent density. EQE measurements were performed using Newport system (QuantX-300) with monochromatic light and white bias light (0.2 Sun). The system was calibrated by a certified silicon solar cell (Newport) each time before the EQE measurement.

### Femtosecond transient absorption measurements

Femtosecond laser pulses were produced using a regeneratively amplified Yb:KGW laser at a 5 kHz repetition rate (Light Conversion, Pharos). The pump pulse was generated by passing a portion of the 1030-nm probe pulse through an optical parametric amplifier (Light Conversion, Orpheus) with the second harmonic of the signal pulse selected for 400 nm light. Both the pump and probe pulses (pulse duration 250 fs) were directed into an optical bench (Ultrafast, Helios), where a white light continuum was generated by focusing the 1030-nm probe pulse through a sapphire crystal. The time delay was adjusted by optically delaying the probe pulse, with time steps increasing exponentially. A chopper was used to block every other pump pulse, and each probe pulse was measured by a charge-coupled device (CCD) after dispersion by a grating spectrograph (Ultrafast, Helios). Samples were prepared on a glass substrate and translated at 1 mm s^−1^ during measurement. Pump fluences were kept at 4 µJ cm^−2^.

### Steady-state PL and time-resolved PL measurements

Steady-state PL and time-resolved PL were measured using a Horiba Fluorolog time-correlated single-photon counting system with photomultiplier tube detectors. Light was illuminated from the top surface of the perovskite film. For steady-state PL measurements, the excitation source is a monochromated Xe lamp (peak wavelength at 520 nm with a line width of 2 nm). For time-resolved PL, we used a green laser diode (*λ* = 540 nm) as the excitation source, with an excitation power density of 5 mW cm^−2^. The PL decay curves were fitted with biexponential components to obtain a fast and a slow decay lifetime. The mean carrier lifetimes τ for the biexponential fit were calculated by the weighted average method.

### Impedance spectroscopy

The impedance spectrum was measured using a potentiostat/galvanostat (AUT50690, PGSTAT204, the Netherlands) at different biases (from −0.20 to 1.05 V). The frequency ranges from 1 MHz to 0.01 Hz with 100 data points. The eigen-relaxation time or recombination time τ was fitted by using *R*_rec_*C*_rec_ (see equivalent circuit in Supplementary Fig. 18). Warburg impedance (*W*_s_) is added to the equivalent circuit due to ion migration in the perovskite, which is manifested as a semi-infinite circle at the low-frequency part (below 10 Hz). The density of the defect states was derived by the angular frequency-dependent capacitance using the equation: *N*_t_(*f*) = −(*V*_bi_ − *V*_app_)/*qW*KT*(d*C*/d*f*)*f, where *V*_bi_, *W*, and *V*_app_ stand for the build-in voltage, the width of the space charge region, and applied voltage, respectively. Then, the *x*-axis was turned from frequency to energetic distance by *E*_a_ = *E*_t_ − *E*_x_, where *E*_x_ is *E*_c_ or *E*_v_, using thermal admittance spectroscopy. Finally, we have the energetic defect distribution under different applied bias voltages as the equation: *N*_t_(*E*_a_) = −(*V*_bi_ − *V*_app_)/*qW*KT*(d*C*/d*f*)*f. For the identification of the energy levels of defects (*E*_a_), we adopted the conversion parameter between the frequency and the energy level, considering the same device architecture and contacting layer^53^. At *T* = 300 K, *E*_a_ = 0.45-0.025ln(2π*f*) in eV.

### Additional characterization

High-resolution SEM images were obtained using the Hitachi SU8230 microscope with an accelerating voltage of 1 kV. A low accelerating voltage and a low beam current were deployed to reduce surface damage of perovskite films under electron beam bombardment. XRD patterns were collected using a Rigaku MiniFlex 600 diffractometer equipped with a NaI scintillation counter and monochromatized Copper Kα radiation (*λ* = 1.5406 Å). Optical absorption measurements were carried out in a Lambda 950 UV/Vis spectrophotometer with an integration sphere. The ratio of MA and FA cations was quantified using ^1^H nuclear magnetic resonance (NMR, Agilent DD2 500), in which 0.5 mL of solution was mixed with 0.1 mL of deuterated water (D_2_O) and 0.02 μL of dimethyl sulfoxide (DMSO) was added as an internal standard. The one-dimensional ^1^H spectrum was measured with water suppression using a pre-saturation method. Quasi-elastic neutron scattering (QENS) measurements were done with the cold neutron chopper spectrometer (CNCS) at the Spallation Neutron Source (SNS) of the Oak Ridge National Laboratory. An incident neutron beam with an energy of 3.32 meV was used. This energy yielded an elastic resolution of 102 μeV and *Q* measurement ranges of 0–2.5. Perovskite single crystals were first ground into powders. The powders were then loaded under helium into cylindrical aluminum cans. Integration of spectra was for |*Q*| = 0.4–1.8. An empty aluminum container was used for background subtraction.

### Computational methods

We performed density functional theory (DFT) calculations using the Vienna Ab Initio Simulation Package (VASP) code^54,55^, wherein a planewave basis set was used under period boundary conditions. The exchange-correlation functional that was employed was the Perdew–Burke–Ernzerhof (PBE)^56^ generalized-gradient approximation and the projector-augmented wave (PAW) method^57^ for ion–electron interactions in the periodic system was used. The planewave kinetic energy cutoff was fixed at 400 eV, and the van der Waals interactions were modeled using DFT-D2 scheme of Grimme^58^. A supercell consisting of 108 ABX_3_ units was employed in all calculations. Here, A can be a mixture of MA, FA, and Cs, and X can be I or Br. For the CsFA configuration, the molar ratio of Cs:FA is 21:87 in order to be as close as possible to the experimental composition that gives a bandgap around 1.7 eV. To model the configuration of the CsMAFA perovskite, we partially replaced Cs with MA to have the molar ratio Cs:MA:FA = 5:16:87 and kept the Br content the same as in the CsFA perovskite. For both CsFA and CsMAFA perovskites, the molar ratio of I:Br is 2:1. The calculated optimized geometries with disordered orientation of organic molecules (cations) for bulk CsFA and CsMAFA perovskites without defects are presented in Supplementary Fig. 13. The same setup was used for the bulk CsFA and CsMAFA perovskites with defects.

The Brillouin zone was sampled using a single (Gamma) k-point, and electronic convergence criterion of 10^−7^ eV per formula unit was used. The computational cells were initially obtained using periodic repetition of unit cells and were then heated to 300 K using NVT molecular dynamics simulations to obtain random orientations of organic molecules. After 3-ps simulations using 1-fs time step, structures were cooled down to 0 K and were relaxed using a conjugate gradient algorithm until the energy converged within 10^−5^ eV per formula unit. The reason that we did not apply the hybrid DFT functionals (i.e., HSE) or the spin orbital coupling (SOC) calculations is to closely relate to the experimental molar ratio of CsMAFA and CsFA perovskite materials; the system will run into memory issue (we were using BGQ-scinet supercomputer, which consist 1024 CPU with 64 nodes per core) when running both HSE and SOC calculations. DFT–GGA calculations without SOC effects were shown to capture semi-quantitative behavior. Good agreement of the DFT bandgap between experiment and theory is largely attributed to large error cancelation^49^.

To examine the effects of organic cation orientation on the defect states of CsMAFA perovskites (as presented in Fig. 4), we fixed one single MA cation close to the defect site with certain orientation and relaxed the rest of the cell to obtain an optimized structure. Due to the large computational cells and low geometry, there is an extremely large number of possible ways to arrange MA cations. Thus, we only chose a single MA cation arrangement to draw our qualitative conclusions. We also note that in while calculating the density of states (as presented in Fig. 4), the Brillouin zone of the supercell was sampled by a finer Monkhorst–Pack mesh, corresponding to a grid of 2 × 2 × 1 k-points. The wave functions (partial charge density of electrons) of the trap states in CsFA and CsMAFA perovskites with defects were calculated by specifying the energy range of the bands using standard settings in the VASP code.

### Data availability

All data that support the findings in this study are present in the paper and the Supplementary Information. Additional data related to this study are available from the corresponding authors on reasonable request.

## Electronic supplementary material

Supplementary Information
