# Supplementary material for: Dipolar cations confer defect tolerance in wide-bandgap metal halide perovskites
Source: Nat Commun. 2018 Aug 6;9:3100. doi: 10.1038/s41467-018-05531-8 (PMC6079062; doi:10.1038/s41467-018-05531-8)
Supplement: Supplementary file 1 — Supplementary Information [file 41467_2018_5531_MOESM1_ESM.pdf]

Supplementary Information for

Dipolar cations confer defect tolerance in wide-bandgap metal halide perovskites

Hairen Tan[1,2†*], Fanglin Che[1†], Mingyang Wei[1†], Yicheng Zhao[1], Makhsud I. Saidaminov[1], Petar Todorovic[1], Danny Broberg[3,4], Grant Walters[1], Furui Tan[1,5], Taotao Zhuang[1], Bin Sun[1], Zhiqin Liang[1], Haifeng Yuan[1,6], Eduard Fron[6], Junghwan Kim[1], Zhenyu Yang[1], Oleksandr Voznyy[1], Mark Asta[3,4], Edward H. Sargent[1*]

[1]Department of Electrical and Computer Engineering, University of Toronto, 35 St. George Street, Toronto, Ontario M5S 1A4, Canada

[2]National Laboratory of Solid State Microstructures, Collaborative Innovation Centre of Advanced Microstructures, Jiangsu Key Laboratory of Artificial Functional Materials, College of Engineering and Applied Sciences, Nanjing University, Nanjing 210093, Jiangsu, China

[3]Department of Materials Science and Engineering, University of California, Berkeley, CA 94720, USA

[4]Materials Sciences Division, Lawrence Berkeley National Laboratory, Berkeley, CA 94720, USA

[5]Key Lab of Photovoltaic Materials, Department of Physics and Electronics, Henan University, Kaifeng, 475004, China

[6]Department of Chemistry, KU Leuven, Celestijnenlaan 200F, B-3001 Leuven, Belgium

[†]These authors contributed equally to this work.

[*]Corresponding authors. E-mail: hairentan@nju.edu.cn (H.T.); ted.sargent@utoronto.ca (E.H.S.)

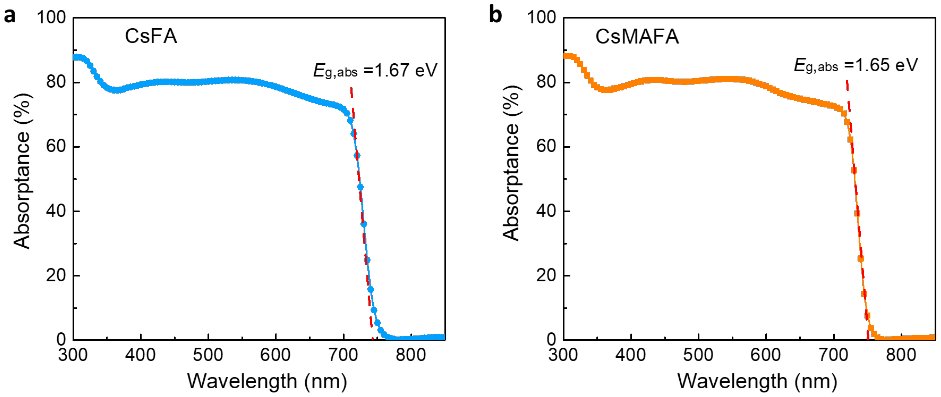

Supplementary Figure 1| Absorption spectra of CsFA (**a**) and CsMAFA (**b**) wide-bandgap perovskite films deposited on glass substrates, exhibiting absorption onsets at 1.67 and 1.65 eV, respectively.

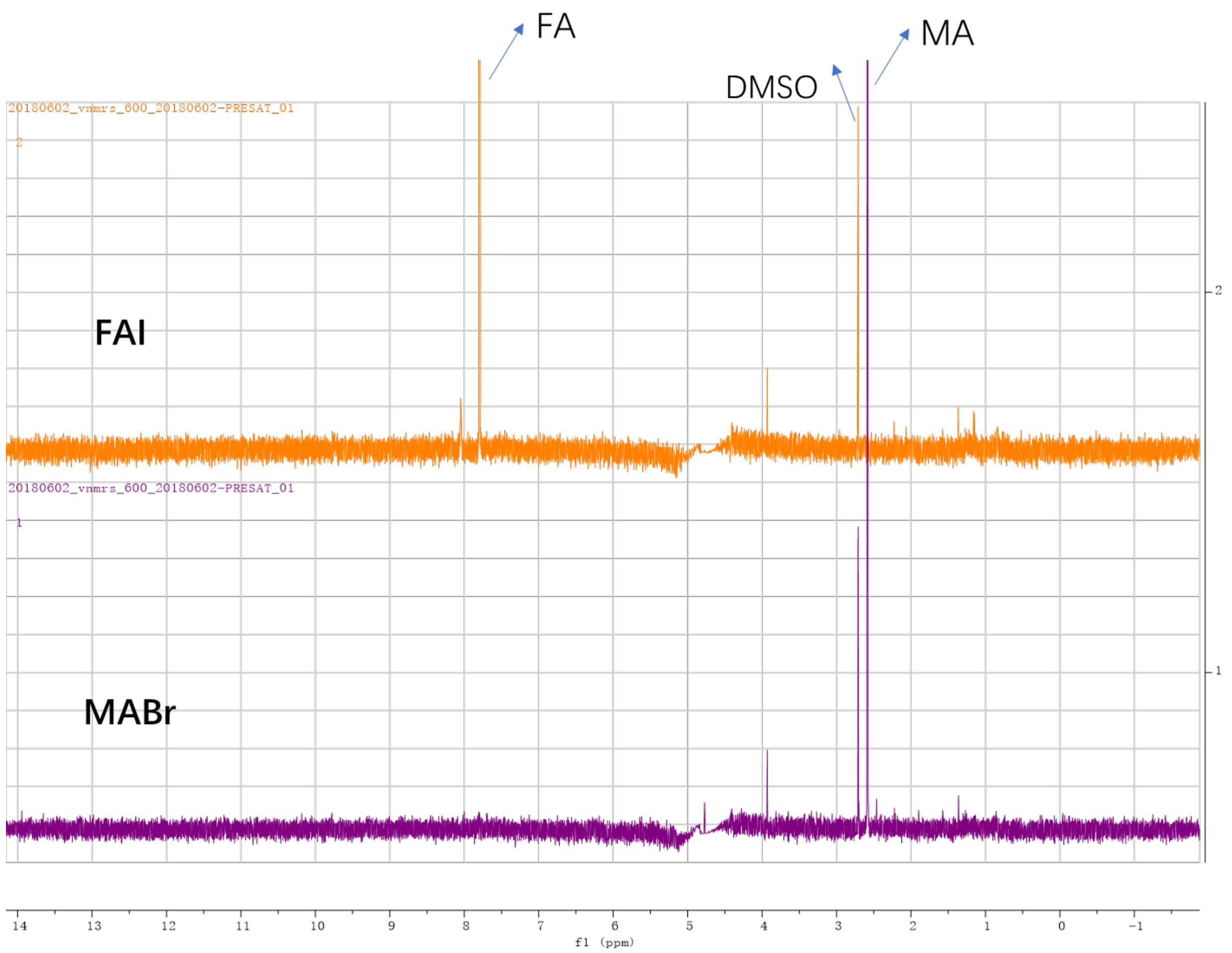

Supplementary Figure 2| NMR spectra of FAI and MABr powders dissolved in deionized water. The FAI and MABr powders (dissolved in deionized water) were measured separately to determine the signals of FA and MA cations.

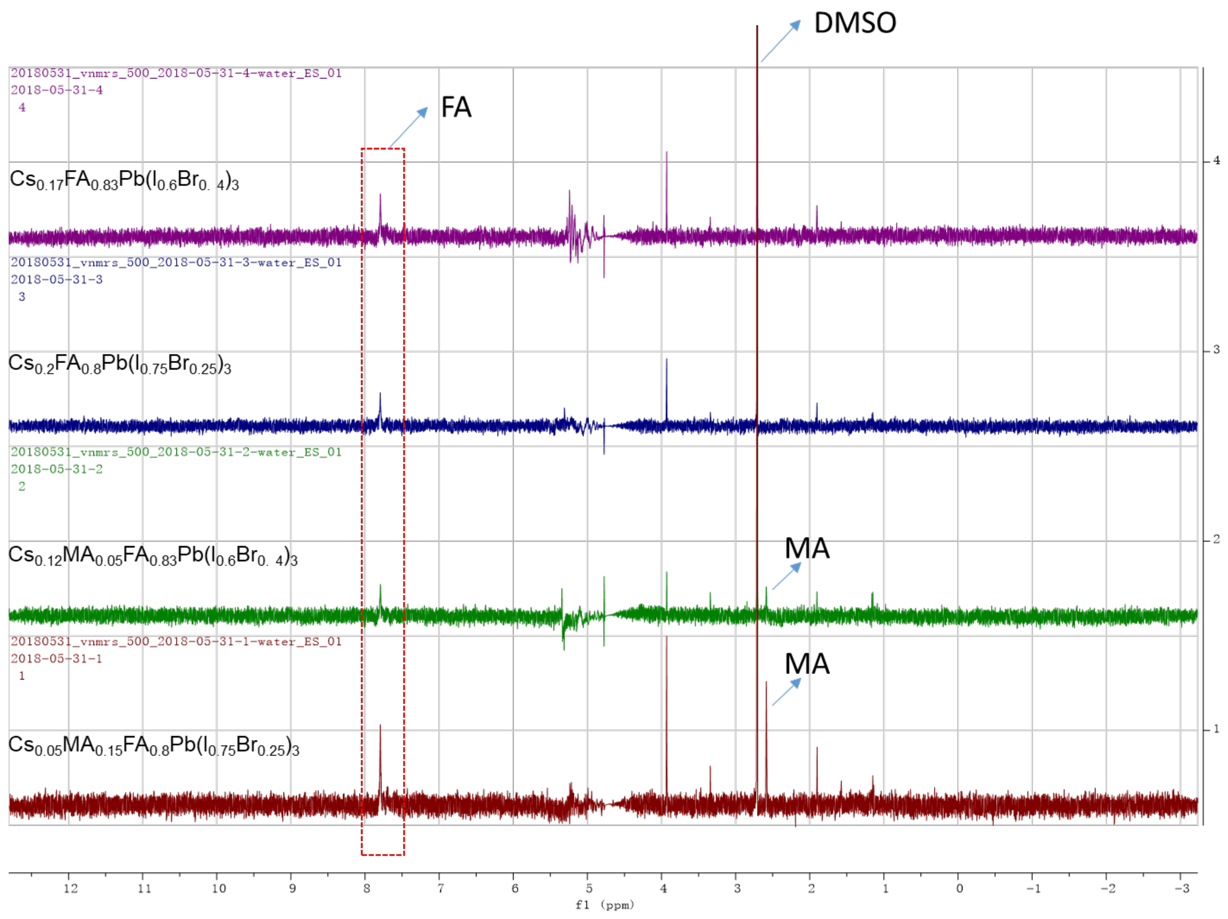

Supplementary Figure 3| NMR spectra of perovskite films dissolved in deionized water. The perovskite films were scratched into powders from the glass substrates. The perovskite powders were then dissolved in deionized water for NMR measurements.

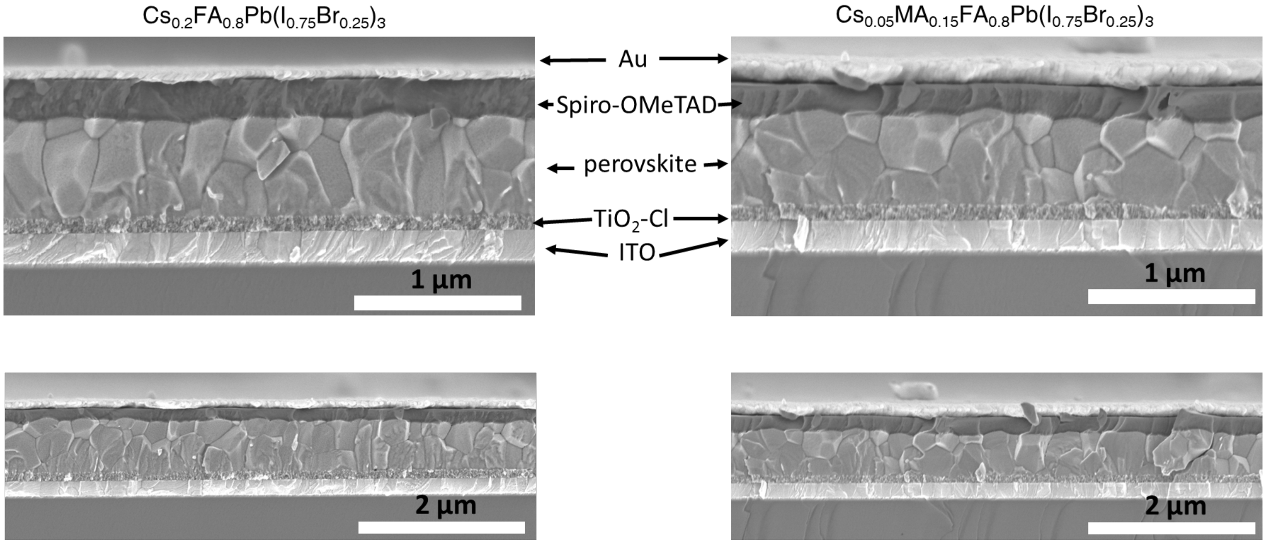

Supplementary Figure 4| Device structure and cross-sectional SEM images of wide-bandgap perovskite solar cells. The perovskite films are dense and smooth for both compositions. The thickness of the perovskite absorber layer is around 600 nm.

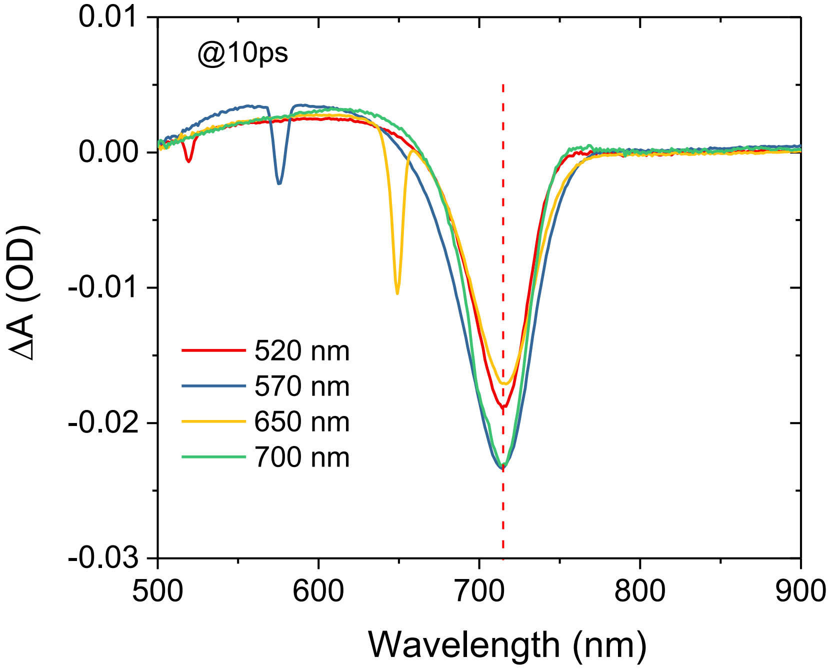

Supplementary Figure 5| Transient absorption spectra of CsMAFA perovskite film on glass substrate at probe delay time of 10 ps under various excitation wavelengths.

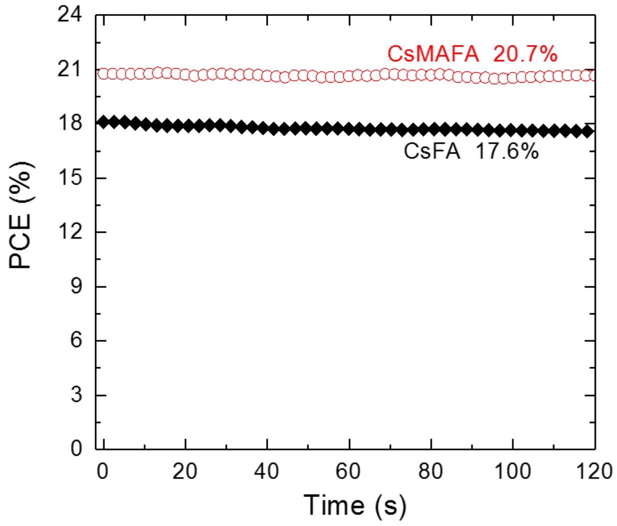

Supplementary Figure 6| Stabilized PCEs of CsFA and CsMAFA perovskite solar cells.

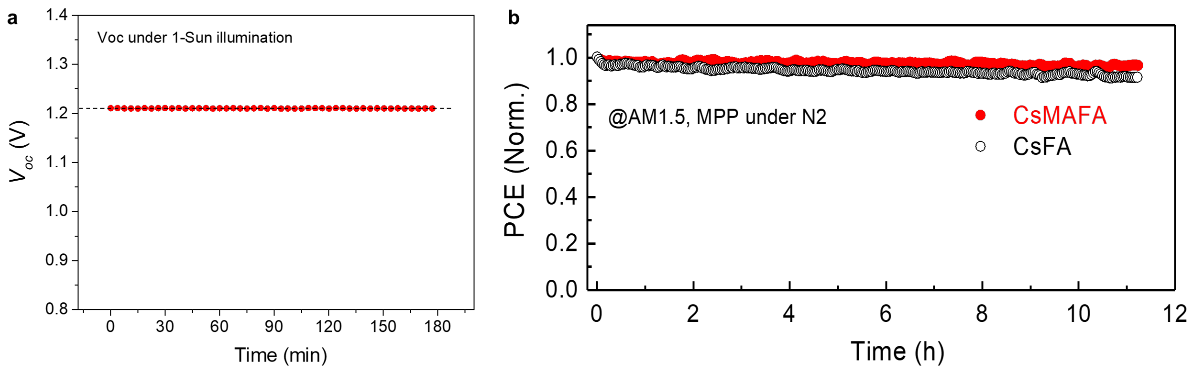

Supplementary Figure 7| a Continuous V_{oc} tracking of a CsMAFA perovskite solar cell under one-sun illumination and operating in a nitrogen environment. **b** Continuous MPP tracking of CsFA and CsMAFA perovskite solar cells under one-sun illumination and operating in a nitrogen environment with a 420-nm UV cut-off filter.

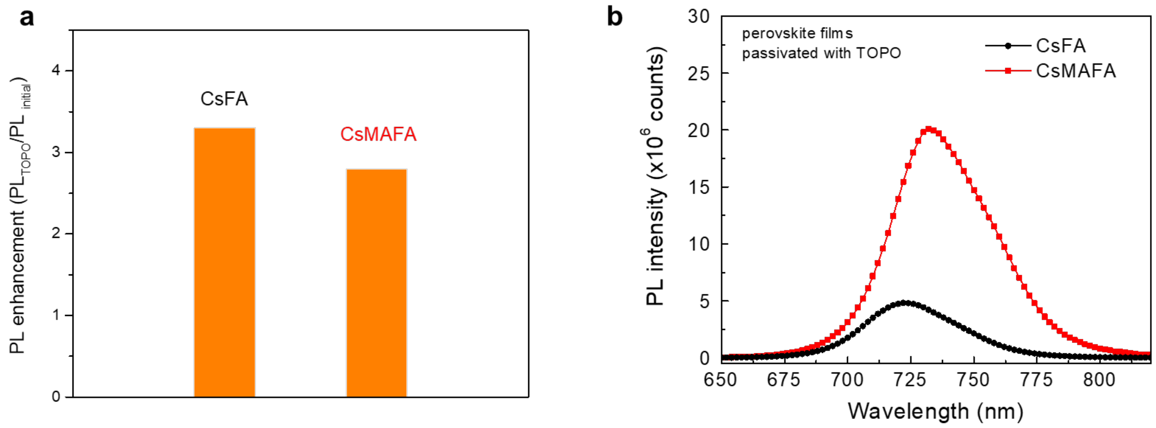

Supplementary Figure 8| PL emission enhancement factor (**a**) and steady-state PL spectra (**b**) of CsFA and CsMAFA perovskite films after TOPO enabled surface/grain passivation. The TOPO treatments were carried out by spin-casting a thin layer of TOPO ligands (0.025 M in anhydrous chlorobenzene) on the as-deposited perovskite films.

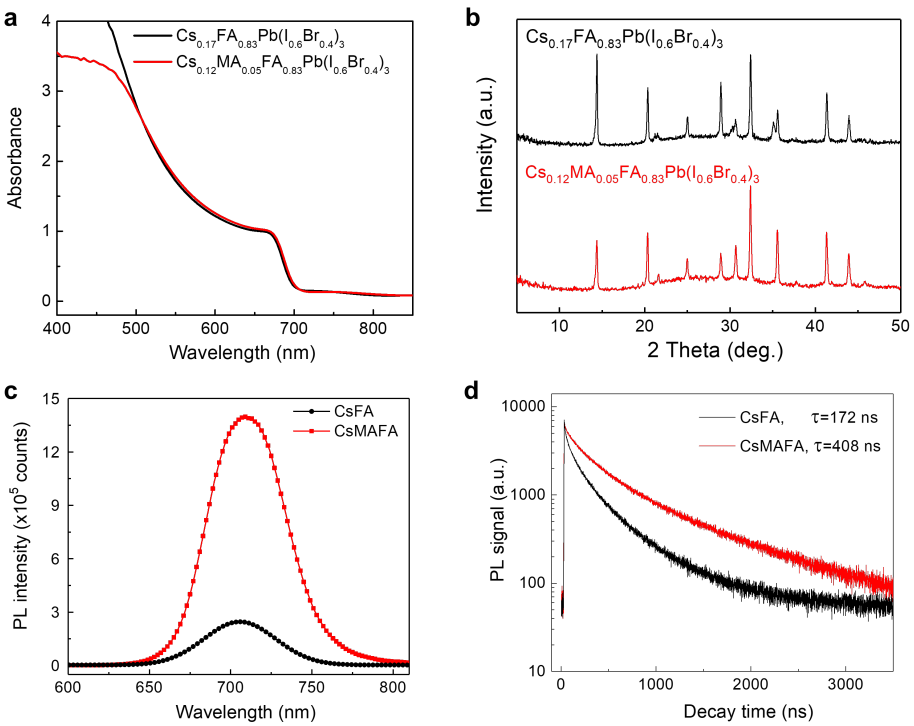

Supplementary Figure 9| Optical and structural characterizations of 1.74-eV-bandgap perovskite films. a Absorbance spectra of CsFA and CsMAFA perovskite films, showing optical absorption onset of 1.74 eV. **b** XRD patterns of perovskite films. Steady-state PL spectra (**c**) and time-resolved PL decay curves (**d**) of CsFA and CsMAFA perovskite films. Here the CsFA and CsMAFA stand for the compositions of $Cs_{0.17}FA_{0.83}Pb(I_{0.6}Br_{0.4})_3$ and $Cs_{0.12}MA_{0.05}FA_{0.83}Pb(I_{0.6}Br_{0.4})_3$, respectively.

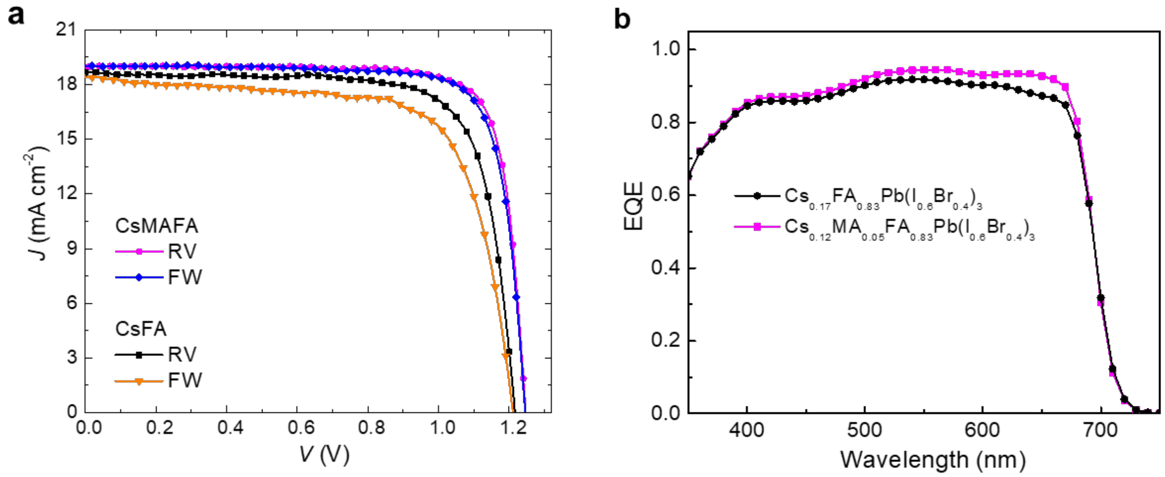

Supplementary Figure 10| *J-V* and EQE curves of best-performing 1.74-eV-bandgap perovskite solar cells. Here the CsFA and CsMAFA stand for the compositions of $Cs_{0.17}FA_{0.83}Pb(I_{0.6}Br_{0.4})_3$ and $Cs_{0.12}MA_{0.05}FA_{0.83}Pb(I_{0.6}Br_{0.4})_3$, respectively.

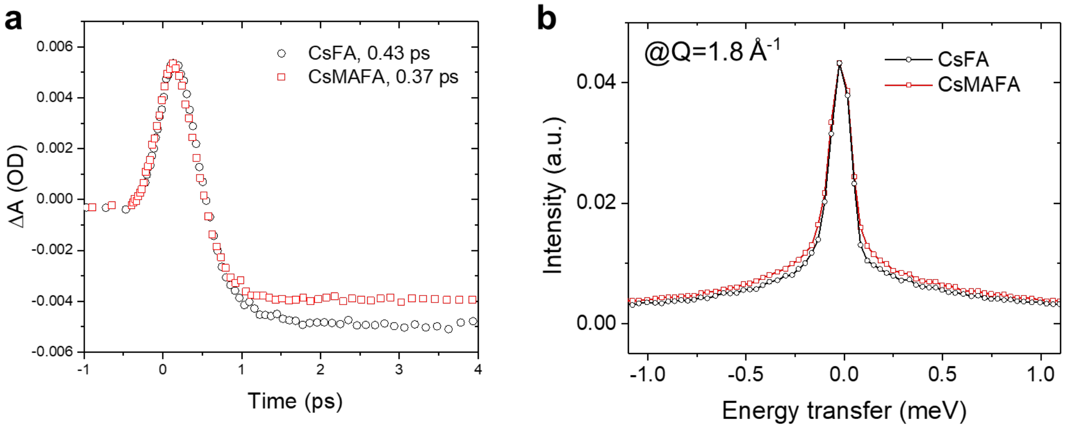

Supplementary Figure 11| Transient absorption and neutron scattering of CsFA and CsMAFA perovskites. a ynamics of screening extracted from transient absorption probed at 740 nm for CsFA and at 750 nm for CsMAFA as a function of pump-probe delay. The indicated polaron formation times are 0.43 and 0.37 ps for CsFA and CsMAFA, respectively. Transient absorption spectroscopy was used here to probe the dynamics of photon-generated free carriers. Figs. 1e-f show a red-shift in the band-edge absorption at delay time shorter than 1 ps for both CsFA and CsMAFA perovskite films. The red-shifted band-edge is attributed to the many-body interactions among generated free carriers. After 1 ps, the red-shifted feature disappears as a result of carrier screening caused by polaron formation. By fitting the decay time of photon-induced absorption (PIA), decay rates of 0.43 ps and 0.37 ps are obtained for FACs and FAMACs perovskite, suggesting their polaron formation rate is essentially the same. **b** Representative quasi-elastic neutron scattering (QENS) measurements of CsFA and CsMAFA perovskites indicating scattered intensity against energy transfer Q measured at 300 K plotted. Here the CsFA and CsMAFA stand for the compositions of $Cs_{0.2}FA_{0.8}Pb(I_{0.75}Br_{0.25})_3$ and $Cs_{0.05}MA_{0.15}FA_{0.8}Pb(I_{0.75}Br_{0.25})_3$, respectively.

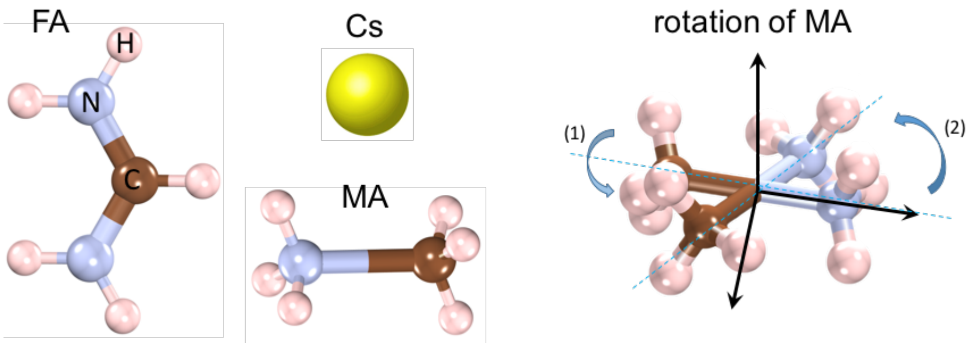

Supplementary Figure 12| Molecule configurations of MA, FA, and Cs cations. The dynamic motion of MA cation can be two ways: (1) methyl and/or ammonium rotation along the C-N axis (with fixed C and N positions) which does not change the local polarization direction; (2) the whole cation reorientation via rotation of the C-N axis itself which correspondingly changes the local polarization direction.

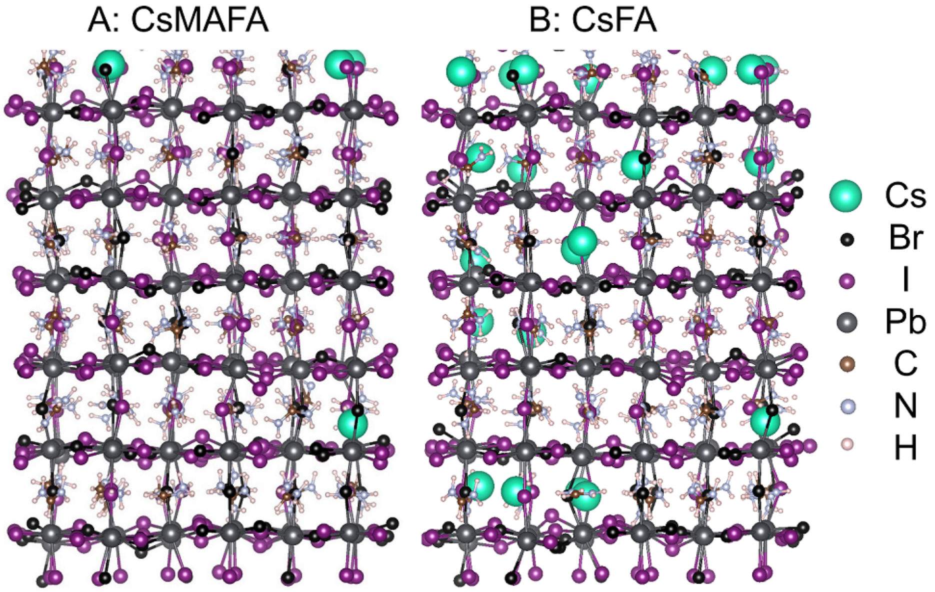

Supplementary Figure 13| The optimized geometries of perovskites for the DFT calculations. a CsMAFA, and **b** CsFA.

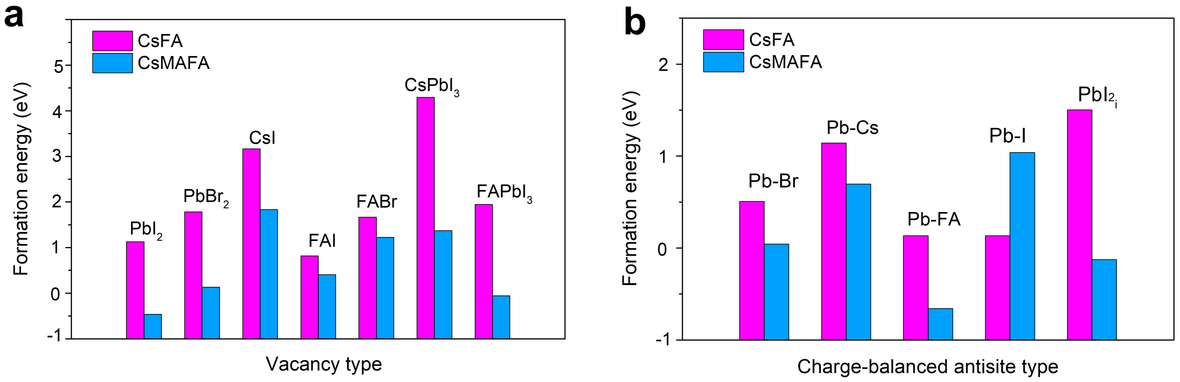

Supplementary Figure 14| The defect formation energy in CsFA and CsMAFA perovskites.
a Schottky-type vacancy defects, and **b** charge-balanced antisite defects.

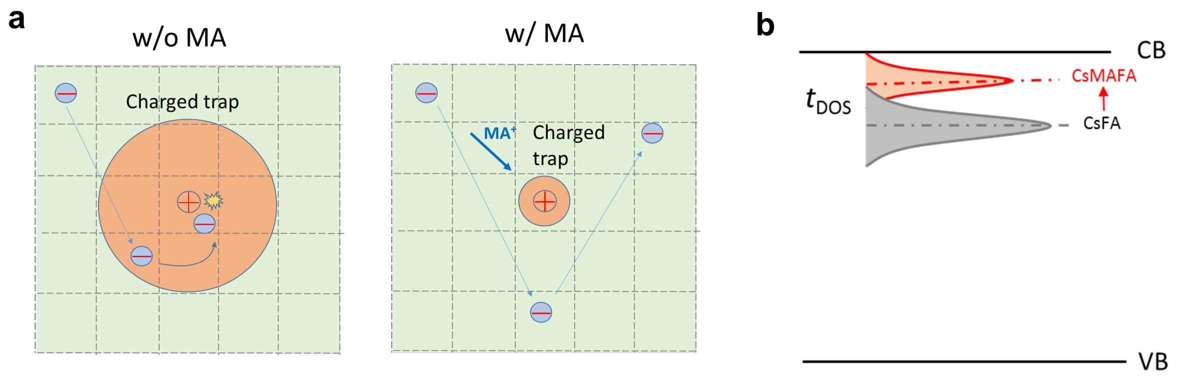

Supplementary Figure 15| Mechanisms of increased defect tolerance in MA-containing perovskite. a Schematic illustration of the charge transport near a trap site in the cases of trap screening with MA (smaller trapping cross-section) and no trap screening in the absence of MA (larger trapping cross-section). The dash square indicates the unit cell of the perovskite crystal. **b** Schematic of the trap density of states distribution for CsFA and CsMAFA perovskites. With MA incorporation, the trap density is reduced, and the trap energy levels shift closer to the CBM or VBM.

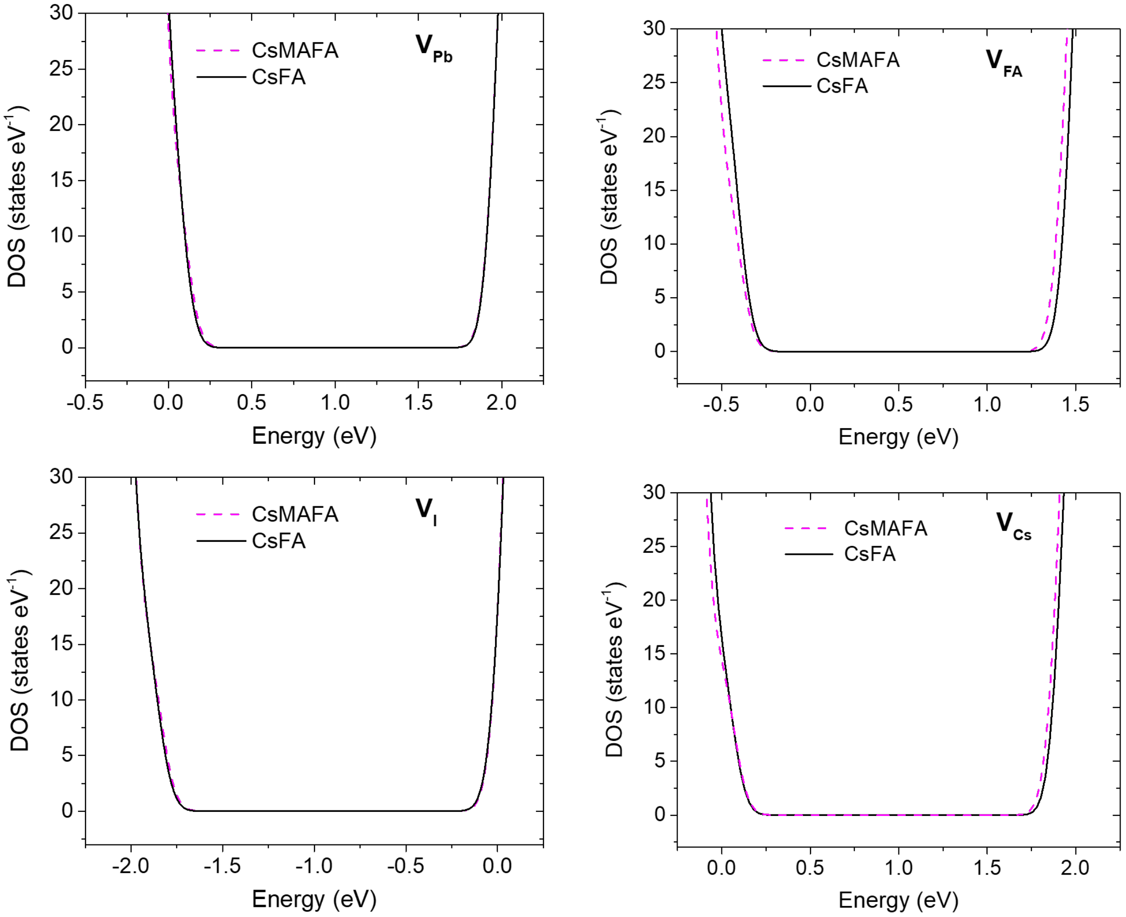

Supplementary Figure 16| DFT calculations investigating the electronic properties of vacancy defects (V_{FA}, V_{Pb} and V_I, and V_{Cs}) in CsMAFA (red dashed line) and CsFA (black solid line) perovskites.

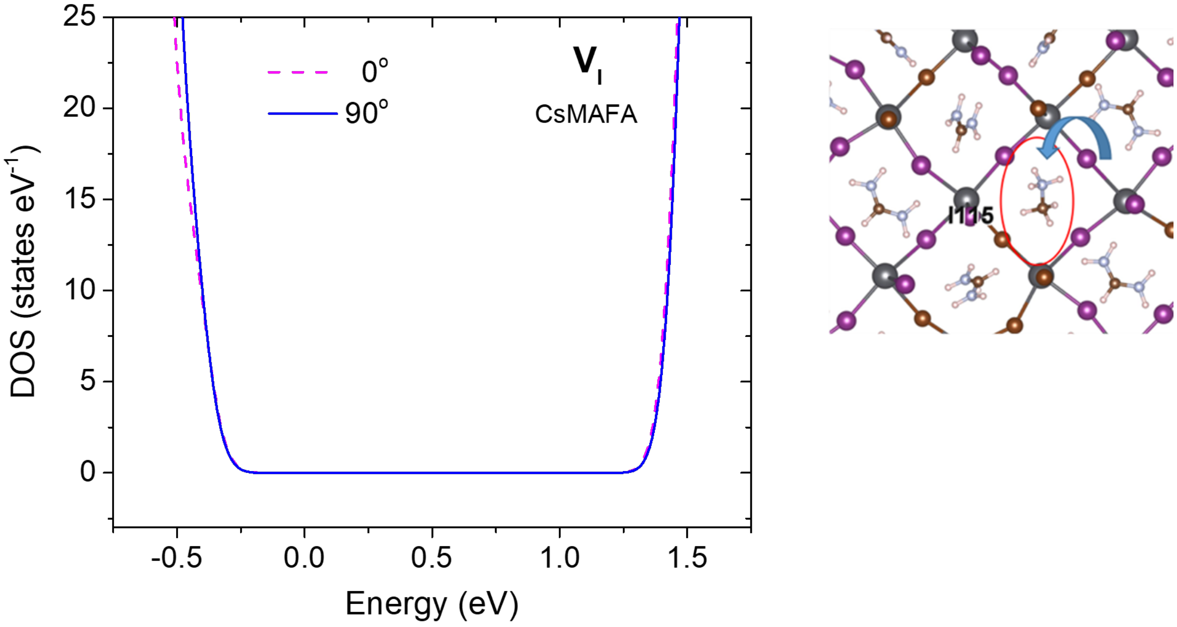

Supplementary Figure 17| DFT calculations investigating the effects of MA reorientation on the electronic properties of I vacancy (V_I) defect in CsMAFA perovskite. The reorientation of MA did not change the electronic properties of the defect.

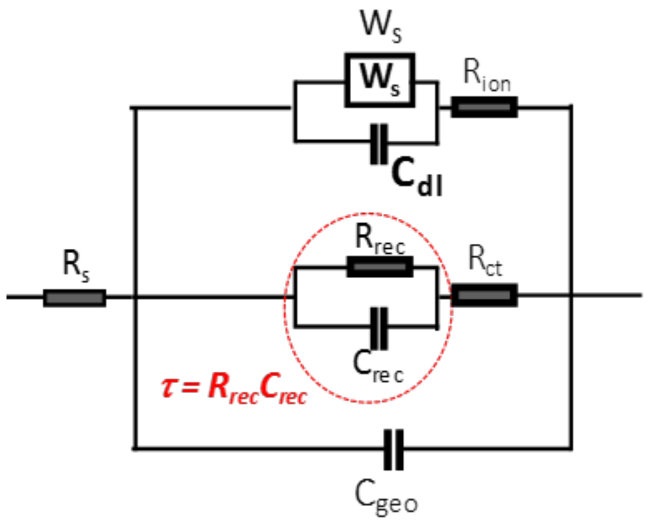

Supplementary Figure 18| Equivalent circuit used for the fitting of impedance spectra to extract the recombination lifetime.

Supplementary Table 1. **Bandgaps of perovskite films determined from absorption onset, Tauc-plot, and PL peaks.** $E_{g,abs}$: absorption onset; $E_{g,Tauc}$: optical Tauc-plot; $E_{g,PL}$: PL emission peak.

**Composition**	$E_{g,abs}$ (eV)	$E_{g,Tauc}$ (eV)	$E_{g,PL}$ (eV)
$Cs_{0.2}FA_{0.8}Pb(I_{0.75}Br_{0.25})_3$	1.67	1.70	1.71
$Cs_{0.05}MA_{0.15}FA_{0.8}Pb(I_{0.75}Br_{0.25})_3$	1.65	1.68	1.69
$Cs_{0.17}FA_{0.83}Pb(I_{0.6}Br_{0.4})_3$	1.74	1.78	1.76
$Cs_{0.12}MA_{0.05}FA_{0.83}Pb(I_{0.6}Br_{0.4})_3$	1.74	1.78	1.75

Supplementary Table 2. The molar ratio of organic cations in the precursor solutions and in the corresponding perovskite films.

**Nominated composition**	**MA/(MA+FA) ratio in precursor solution**	**MA/(MA+FA) ratio in perovskite film**
$Cs_{0.2}FA_{0.8}Pb(I_{0.75}Br_{0.25})_3$	0	**0**
$Cs_{0.05}MA_{0.15}FA_{0.8}Pb(I_{0.75}Br_{0.25})_3$	0.16	**0.17**
$Cs_{0.17}FA_{0.83}Pb(I_{0.6}Br_{0.4})_3$	0	**0**
$Cs_{0.12}MA_{0.05}FA_{0.83}Pb(I_{0.6}Br_{0.4})_3$	0.06	**0.07**

Supplementary Table 3. The total energies (in eV) of the interstitial defect Pb_i, the antisite defect I_{Pb}, and the antisite defect Br_{Pb} in CsMAFA perovskite with various MA orientations. For each defect type, the energies are referred to the most stable MA orientation case (0.00 for formation energy).

Defects	MA 0°	MA 90°	MA 180°	MA 270°
Pb_i	0.43	0.00	0.34	0.43
I_{Pb}	0.95	0.22	0.00	0.91
Br_{Pb}	0.50	0.29	0.00	0.51

Supplementary Note 1:

Formation energy of Schottky-type vacancy defects and charge-balanced antisite Defects

In this study, we have examined various defects in both CsMAFA and CsFA systems, including Schottky defects, vacancies, antisite and interstitial defects. For the Schottky-type defects, we considered the partial Schottky defects of CsI, FAI, PbI_2, and the full Schottky defects of $FAPbI_3$. The formation energies of Schottky defects are calculated by:

$$E^f_{\mathrm{PbI_2}} = E_{\mathrm{V_{Pb^{2+}}}} + 2E_{\mathrm{V_{I^-}}} + E_{\mathrm{PbI_2}} - E_{\mathrm{no\_defect}}$$

$$E^f_{\mathrm{CsI}} = E_{\mathrm{V_{Cs^+}}} + E_{\mathrm{V_{I^-}}} + E_{\mathrm{CsI}} - E_{\mathrm{no\_defect}}$$

$$E^f_{\mathrm{FAI}} = E_{\mathrm{V_{FA^+}}} + E_{\mathrm{V_{I^-}}} + E_{\mathrm{FAI}} - E_{\mathrm{no\_defect}}$$

$$E^f_{\mathrm{FAPbI_3}} = E_{\mathrm{V_{FA^+}}} + 3E_{\mathrm{V_{I^-}}} + E_{\mathrm{V_{Pb^{2+}}}} + E_{\mathrm{FAPbI_3}} - E_{\mathrm{no\_defect}}$$

where $E_{\mathrm{V_{Pb^{2+}}}}$, $E_{\mathrm{V_{Cs^+}}}$, $E_{\mathrm{V_{FA^+}}}$, $E_{V_{I^-}}$ stand for total energies for the perovskite material with vacancies of Pb^{2+}, Cs^+, FA^+ and I^-, respectively. $E_{\mathrm{PbI_2}}$, E_{CsI}, E_{FAI} and $E_{\mathrm{FAPbI_3}}$are the total bulk energies of PbI_2, CsI, FAI, $FAPbI_3$. $E_{\mathrm{no\_defect}}$ is the total energies of the CsMAFA and CsFA systems without any defect.

We also calculated another defect type - charge balanced antisite defects, which switch the location of A or Pb or X in $APbX_3$ perovskites. In this case, the formation energies of charge-balanced antisite defects are calculated by:

$$E^f = E_{\mathrm{defect}} - E_{\mathrm{no\_defect}}$$

where E_{defect} stands for total energies for the perovskite material with the charge-balanced antisite defect and $E_{\mathrm{no\_defect}}$ is the total energies of the CsMAFA and CsFA bulk systems without any defect.

Intrinsic Point Charged Defects

In this study, we also examined various intrinsic point defects in both CsMAFA and CsFA systems, including vacancies, antisite and interstitial (non-)charged defects. For the vacancy defects, we have studied $\mathrm{V_{Pb^{\delta+}}}$($\delta$ stand for possible charge states of Pb, including charge states of 2, 1, 0), $\mathrm{V_{Cs^{\delta+}}}$, $\mathrm{V_{FA^{\delta+}}}$, $\mathrm{V}_{I^{\delta-}}$, and $\mathrm{V}_{Br^{\delta-}}$ (δ stand for possible charge states of 1 and 0). For the antisite defects, we have studied the defect type of $\mathrm{I_{Pb}}^{\delta-}$ or $\mathrm{Br_{Pb}}^{\delta-}$ (impurity component I (or Br) at Pb site, with the possible charge states of 3, 2, 1, 0) and $\mathrm{Pb_I}^{\delta+}$ or $\mathrm{Pb_{Br}}^{\delta+}$ with the possible charge states of 3, 2, 1, 0. We also studied the defects of $\mathrm{Cs_{Pb}}^{\delta-}$, $\mathrm{FA_{Pb}}^{\delta-}$ or $\mathrm{Pb_{Cs}}^{\delta+}$or $\mathrm{Pb_{FA}}^{\delta+}$ with possible charge states of 1 and 0, respectively. For the interstitial defects, we only examined two different defects: $\mathrm{Pb_i}^{\delta+}$ (with possible charge states of 2, 1 and 0), and $\mathrm{I_i}^{\delta-}$ (with possible charge states of 1 and 0) since they are the most easily formed interstitial defects.
